# Case Report: Halting terminal osseous overgrowth post tibia amputation in children: a report of three cases

**DOI:** 10.3389/fsurg.2024.1320661

**Published:** 2024-05-24

**Authors:** Chee-Chun Pon, Ting-Jie Ong, Ahmad Fazly Abd Rasid, Abdul Halim Abd Rashid, Kamal Jamil

**Affiliations:** Department of Orthopedic & Traumatology, Fakulti Perubatan Universiti Kebangsaan Malaysia, Hospital Canselor Tuanku Muhriz, Universiti Kebangsaan Malaysia, Kuala Lumpur, Malaysia

**Keywords:** osseous overgrowth, osteocartilaginous transfer, pediatric amputation, fibula transfer, trans-diaphyseal amputation

## Abstract

Terminal osseous overgrowth is a common complication after trans-diaphyseal amputation in children, leading to pain, soft tissue problems, and recurrent surgical procedures. We report three different cases with post-amputation issues of osseous overgrowth, ulceration, and deformity over the amputation site. The first case involves a 9-year-old boy with a right leg congenital amputation secondary to amniotic band syndrome. The right below-knee stump later experienced recurrent episodes of osseous overgrowth, leading to ulceration. After the prominent tibia was resected and capped with the ipsilateral proximal fibula, a positive outcome was achieved with no more recurrent overgrowth over the right leg stump. The second case involves a 9-year-old girl born with an amniotic constriction band over both legs. Her left leg remained functional after a circumferential Z-plasty, but the right leg was a congenital below-knee amputation. Multiple refashioning surgeries were performed on the right leg due to osseous overgrowth but the patient continued to experience recurrent overgrowth causing pain and difficulty fitting into a prosthesis. We performed osteocartilaginous transfer of the proximal part of the ipsilateral fibula to the right tibial end, successfully preventing the overgrowth of the tibia without any complications. The third case involves an 11-year-old boy with a history of meningococcal septicemia who underwent a right below-knee amputation and left ankle disarticulation due to complications of septic emboli. He experienced a prominent right distal tibia stump, which later developed into valgus deformity as a result of the previous insult to the proximal tibial growth plate. We performed a corrective osteotomy over the proximal right tibia and capped the entire tibia with the ipsilateral fibula as an intramedullary splint for the osteotomy site. Post-operatively, we achieved satisfactory deformity correction and successfully halted the recurrent overgrowth over the right tibia stump. The method of ipsilateral fibula capping is safe and effective in managing the osseous overgrowth complications in trans-diaphyseal amputations among children. Therefore, it is a reasonable option during primary below-knee amputations in children compared to multiple refashioning surgeries.

## Introduction

Osseous overgrowth is a common complication after trans-diaphyseal amputations in children under 12 years old (1). Such overgrowth can originate from periosteal, endosteal, or heterotopic sources (2). Periosteal and endosteal overgrowth mainly occur in a cone shape at the stump's tip, often accompanied by a sharp spike. This is followed by local appositional overgrowth resulting from the healing process in children (3, 4). The local appositional growth at the amputation site can lead to problems such as skin ulceration, swelling, erythema, and pain, making it challenging to fit a prosthesis (5). This overgrowth condition is often recurrent, requiring multiple revision surgeries that can negatively impact the quality of life for amputees and expose them to the risks associated with repeated anesthesia administration. Various treatment strategies have been proposed for managing terminal osseous overgrowth in children, including distal resection, modified Ertl tibiofibular osteomyoplasty (6), capping the bony stump with silicone (3) or Teflon (polytetrafluoroethylene) (3), and biological stump capping involving osteocartilaginous caps from the proximal part of the fibular bone or cortical bone from the amputated distal stump or iliac crest (7–10). Marquardt and Corell introduced the use of an autologous osteocartilaginous cap to cover the diaphysis stump post-amputation, aiming to prevent recurrent overgrowth, based on the observation that overgrowth does not occur from the growth plate (9). This capping technique provides the stump with partial end-bearing capacity and a predictable growth pattern. The rate of recurrent terminal osseous overgrowth with autologous stump capping is reported to be 10% (11). Although osseous overgrowth is commonly observed in children following amputations, amputation itself is not a frequently performed procedure for pediatric patients in our center. We present the outcomes of three cases following the free transfer of a non-vascularized proximal fibula autograft to the trans-diaphyseal tibial stump.

## Case presentation

### Patient 1

**Figure 1:**
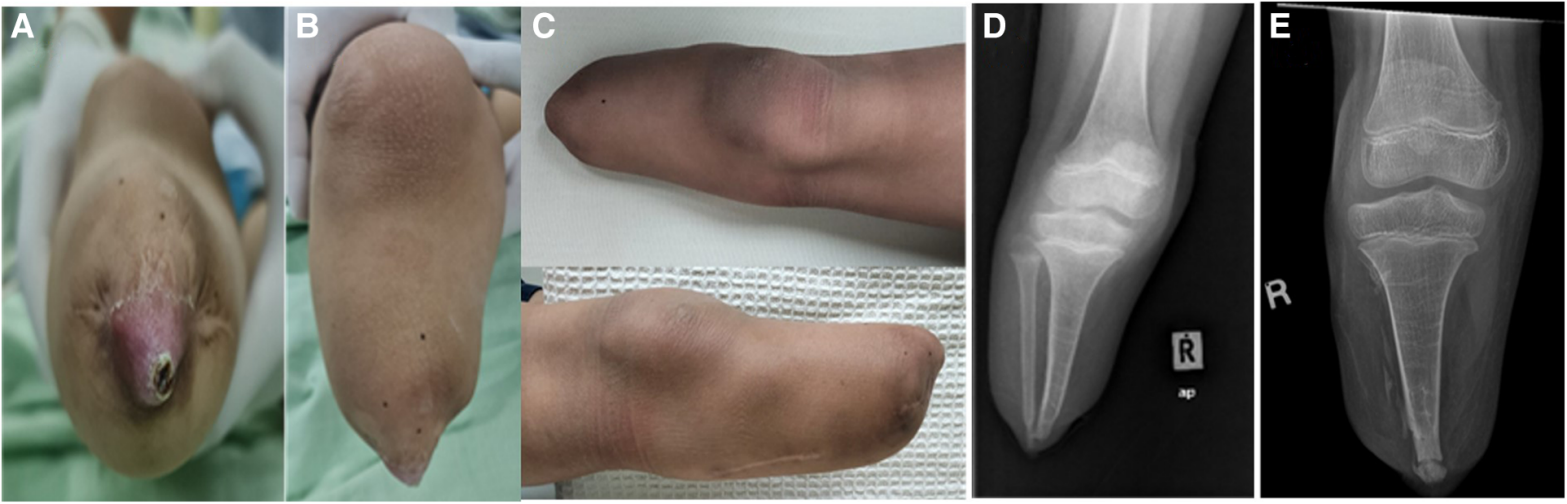
(**A**) and (**B**) show ulceration and bony prominence over the right tibial stump prior to the operation. (**C**) The right tibia stump without any significant osseous growth after 2 years post-operation. (**D**) The pre-operative plain radiographs revealing excessive osseous growth over the distal tibia and fibula, with the growth almost penetrating through the soft tissue. (**E**) exhibits post-operative plain radiographs taken 2 years after the surgery, demonstrating the union of the fibula with the distal tibia and the absence of excessive osseous growth.

A 9-year-old boy who was born with amniotic band syndrome affecting his right leg, which necessitated a transtibial amputation. Over time, the right tibia stump developed osseous overgrowth, causing pain and skin ulceration. Surgical interventions were performed at the ages of 1 year and 6 months, as well as 5 years, which involved resection of the overgrown bone and stump refashioning. However, no distal tibia capping was performed during these procedures. At the age of 8, the patient began to complain of prominent growth and pain in the right tibia stump, along with skin ulceration, which affected his ability to walk and fit a prosthesis. Physical examinations revealed a prominent bone protrusion with ulceration at the end of the right below-knee stump with no signs of infection. Plain radiographs revealed excessive overgrowth of the distal end of the fibula and tibia, almost piercing through the soft tissue. At this point, a surgical revision of the right tibia stump with osteocartilaginous capping using the ipsilateral fibula was proposed to reduce the risk of recurrent osseous overgrowth. During the surgery, dual incisions were made to approach the distal tibia fibula and proximal fibula separately. The periosteum of the distal tibia was released and elevated proximally, followed by the resection of the prominent tibia end (approximately 5 cm) to accommodate the proximal fibula graft. The proximal fibula was exposed and carefully dissected to avoid damage to the common peroneal nerve. The lateral collateral ligament (LCL) and insertion of biceps femoris tendon (BFT) were released from the proximal fibula. The entire fibula was then removed, and soft tissue was trimmed off from the fibula head and metaphysis. The proximal fibula head with metaphysis was inserted into the intramedullary canal of the distal tibia. Fixation was not necessary as there was an interference fit between the fibula and tibia. We did not repair the LCL and BFT but suture them to the surrounding soft tissues. The tibia periosteum was flipped back distally and sutured to the surrounding fibular articular cartilage. Finally, the tibia stump was closed using routine techniques. Following the surgery, the right tibia stump healed without complications, and the patient was able to ambulate comfortably with a well-fitted prosthesis, experiencing no pain or difficulties. No skin ulceration, knee joint instability or terminal osseous overgrowth was observed during the 2-year follow-up period (Figure 1).

### Patient 2

**Figure 2:**
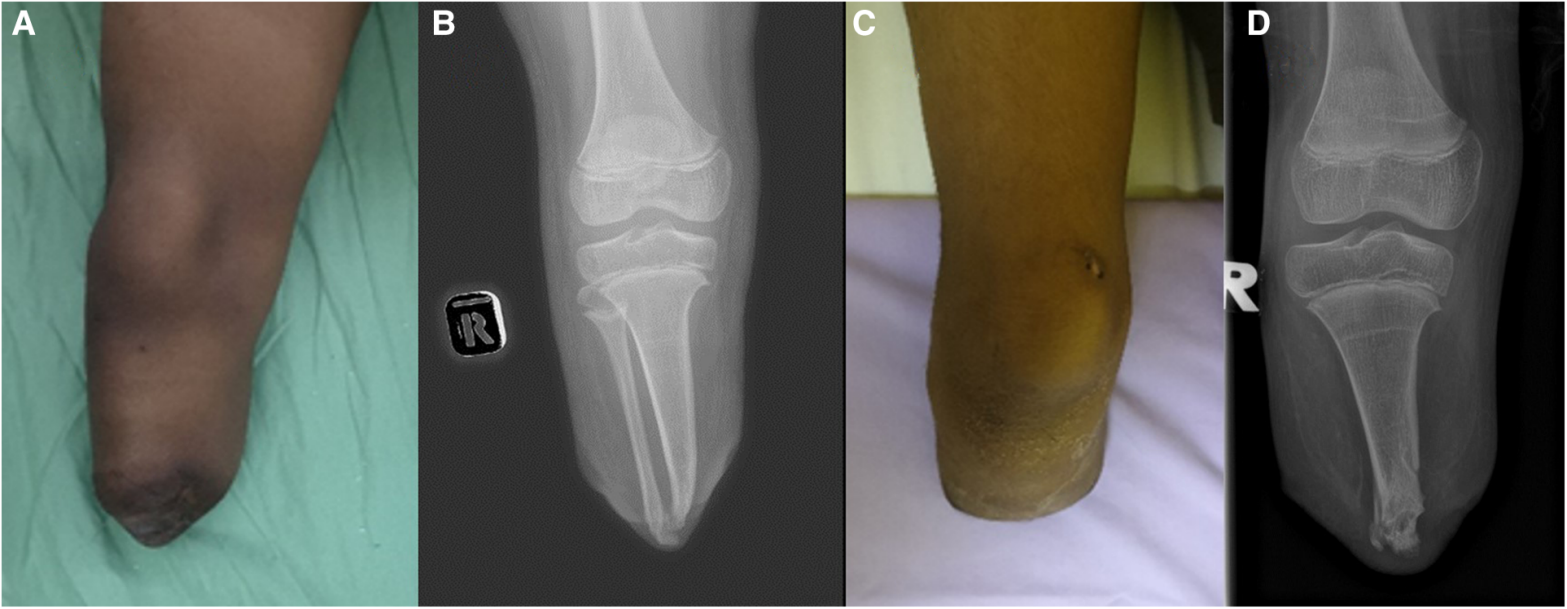
(**A**) Illustrates the prominent distal right below-knee stump. (**B**) showcases the overgrowth of the distal tibia and fibula. (**C**) displays the post-operative image after the ipsilateral fibula transfer to the right knee stump. (**D**) exhibits the corresponding plain radiographs.

A 9-year-old girl who was born with an amniotic constriction band affecting both lower limbs. During infancy, circumferential Z-plasty was performed on her left lower limb, while the right side resulted in a congenital below-knee amputation. Subsequently, the patient underwent a total of five refashioning surgeries due to recurrent overgrowth of her right leg stump. At the latest presentation, the patient complained of overgrowth of her right below-knee amputation stump, which caused pain during ambulation. Physical examination revealed a prominent bone protrusion of approximately 3 cm at the end of the stump, making it difficult to fit into a prosthesis (Figure 2). There were no signs of infection, and blood investigations and infective parameters were within normal ranges. Radiographs of the right below-knee stump showed overgrowth of the distal tibia and fibula. To address the recurrent overgrowth, surgical revisions were performed on the right below-knee stump, involving the transfer of the proximal ipsilateral fibula to the distal tibia. Intraoperatively, dual incisions were made to approach the distal tibia fibula and proximal fibula. Approximately 5 cm of the tibial end was excised, resulting in a remaining stump length of 7 cm from the tibial tuberosity. Through the lateral approach to the fibular head, the ipsilateral fibula was mobilized using a periosteal stripper and harvested. The common peroneal nerve was sharply transacted to allow it to retract into the stump. The proximal part of the fibula was transferred to serve as a tibial intramedullary end-cap. Similar to Patient 1, the LCL and BFT were not repaired. The preserved tibial periosteum was used to cover the joint of the end-cap. The stump end of the elliptical incision was closed in layers. The right below-knee stump wound healed well, and no joint instability or osseous overgrowth was observed during the 2-year follow-up period. The patient was able to ambulate effectively with a well-fitted prosthesis.

### Patient 3

**Figure 3:**
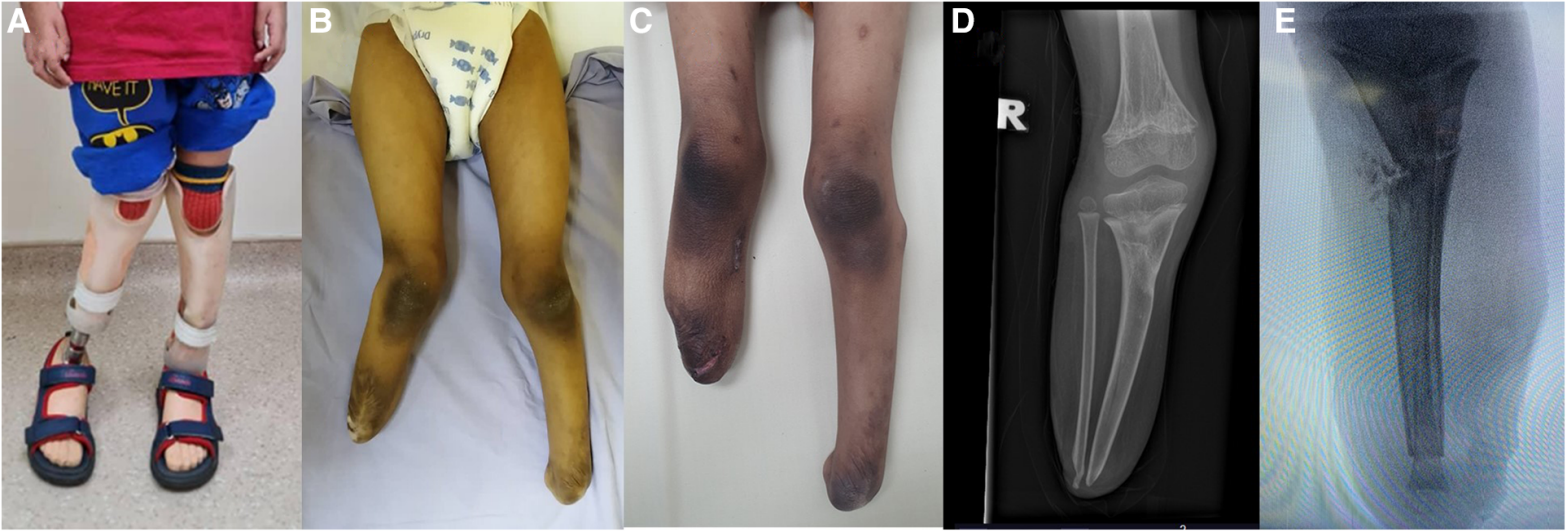
(**A**) and (**B**) depict the patient's initial presentation with valgus deformity of the right knee and a prominent right below-knee stump. (**C**) shows the corrected valgus deformity of the right below-knee stump after the surgery. (**D**) displays preoperative plain radiographs revealing overgrowth of the distal tibia and fibula, as well as valgus deformity at the proximal tibia. (**E**) illustrates the closed wedge osteotomy performed on the proximal right tibia and the intramedullary splint created using the ipsilateral fibula.

**Figure 4:**
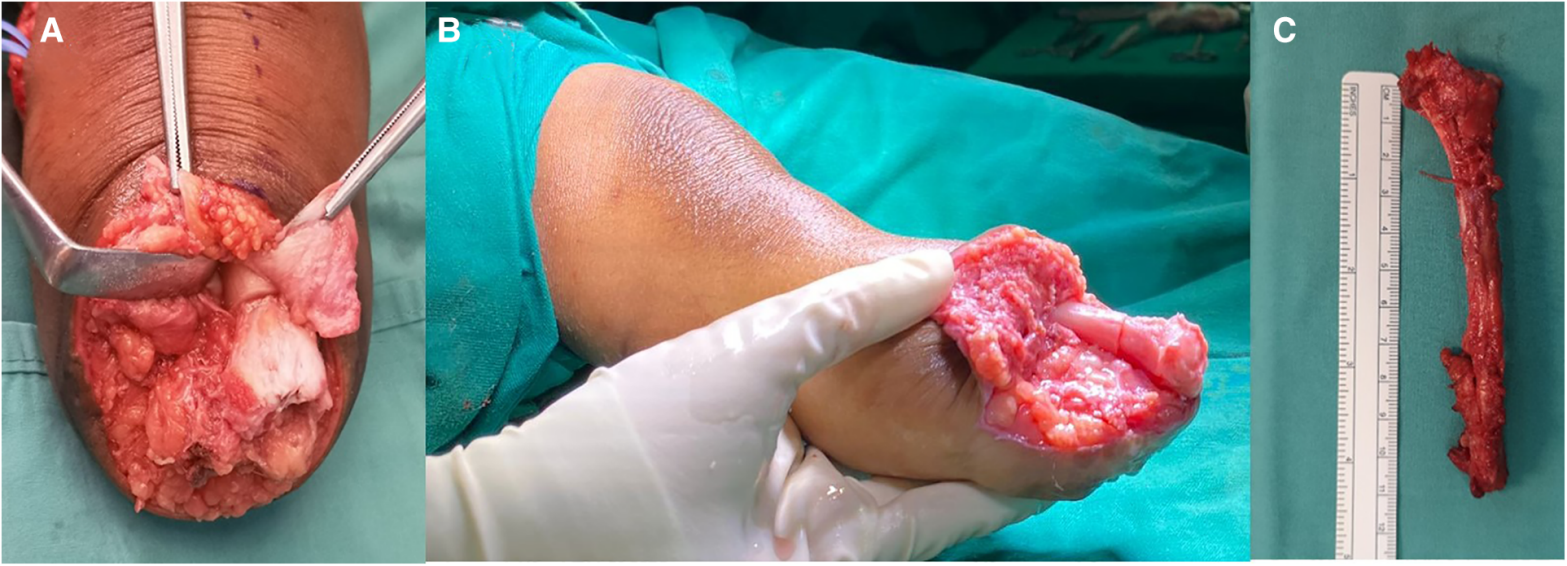
(**A**) and (**B**) are intraoperative photos showing the fibular head with the metaphyseal-diaphyseal end inserted into the tibial medullary canal, acting as an intramedullary end-cap. (**C**) displays the harvested ipsilateral fibula.

An 8-year-old boy with a history of meningococcal septicaemia and purpura fulminans at the age of 2, which resulted in bilateral feet gangrene due to septic emboli. As a result, the patient underwent a right below-knee amputation and left ankle disarticulation to remove the gangrenous feet. Our institution has reported the initial management and the pathological description of the condition previously (12). Following the surgeries, bilateral lower limb prostheses were provided for ambulation. At the age of 5, the patient underwent a right below-knee stump refashioning procedure and proximal tibia epiphysiolysis with fat graft due to overgrowth of the stump. However, after 1 year, the right below-knee stump presented with valgus deformity and prominent osseous growth. The patient experienced difficulties in ambulation and reported pain during prosthesis fitting. Physical examination revealed a prominent distal right knee stump and valgus deformity. Blood parameters were within normal ranges. Plain radiographs indicated a valgus deformity of approximately 20 degrees in the proximal right tibia, along with overgrowth of the distal tibia and fibula. To address both the deformity and overgrowth, corrective osteotomy of the proximal right tibia and transfer of the ipsilateral fibula were performed (Figure 3). Intraoperatively, dual incisions were made over the distal stump and the proximal lateral leg to access the distal tibia fibula and proximal fibula, respectively. Approximately 5 cm of the distal tibia was shortened. The fibula was harvested as a non-vascularized graft and trimmed to remove the overgrown portion. The common peroneal nerve was carefully dissected away from the bone. The insertion of LCL and BFT which were detached from fibular head were then sutured to surrounding tissue. Another incision was made over the proximal medial leg for a closed wedge osteotomy of the tibia to correct the valgus deformity. The entire fibula was reversed and transferred into the tibia's intramedullary canal, acting as an intramedullary splint for the osteotomy site. The fibula was snugly fit with the tibia, eliminating the need for additional fixation (Figure 4). The end cap joint was covered with tibia periosteal tissue, and the stump was closed using standard techniques. Postoperatively, the right below-knee stump healed well, and the deformity was corrected. No joint instability or osseous overgrowth was observed during the 1-year follow-up period. The patient and parents expressed satisfaction with the outcome of the right below-knee stump, as it fit well into the prosthesis and improved the patient's ability to ambulate.

In all three patients, a period of postoperative immobilization was followed by fitting of prosthesis. This includes application of a u-slab cast for a month, except Patient 3 who had a longer period of protection for 3 months duration. The measurement for a new prosthesis was performed once the cast was taken off and the wound had healed well.

## Discussion

Osseous overgrowth is a significant complication following amputation in children, leading to various difficulties and complications. The prevalence of this condition is reported to be as high as 86% (5). It is important to note that stump overgrowth is a localized phenomenon occurring in the distal stump as a result of the healing process, rather than continuous growth from the proximal physis. This suggests that overgrowth is related to the local bone healing process at the amputation site. Studies investigating the histology of stump overgrowth in rabbits have indicated that the medullary canal is the source of this overgrowth (13). This finding explains why overgrowth does not occur in cases of disarticulation where the articular cartilage remains intact, as opposed to cases where the bone is transected. Further research by Speer et al. has shed light on the pathophysiology of stump overgrowth, specifically addressing why it does not occur in the mature skeleton. Their experimental histological study conducted on the immature skeleton of rabbits suggests that the amputation stump responds through wound healing and intramembranous bone formation (4). In a young skeleton, the periosteum has enough elasticity to separate from the end of the amputated limb, resulting in the growth of new bone in that area.

Resection alone as a treatment for stump overgrowth has been found to have a high recurrence rate, reaching up to 85.7% (3). To address this issue and prevent appositional growth from the intramedullary bone end, various methods of capping the medullary canal have been proposed. These methods aim to disrupt the interaction between the endosteum (inner layer of bone) and the surrounding soft and bony tissues. Both synthetic and biological capping techniques have shown positive outcomes in terms of reducing recurrent overgrowth. The use of a biological cap resulted in a recurrence rate of only 14%, while synthetic polytetrafluoroethylene (PTFE) caps showed no recurrence (3). However, it is important to note that while synthetic capping materials can effectively reduce bony growth, they have limitations. The revision rate for synthetic caps is high due to issues such as fixation failure, infection, implant fracture, and difficulties in achieving proper soft tissue coverage. Synthetic caps must be biologically inert and durable to ensure long-term success. Among synthetic materials, Teflon caps have shown better results compared to others, with a revision rate of 29%. Interestingly, the failure of both synthetic and bone graft caps is primarily attributed to factors such as infection and painful bursa, rather than overgrowth itself (3).

Among the biological caps, the osseocartilaginous cap is a popular method due to its low revision rate for recurrent bone overgrowth. Marquardt and Correll utilized an autologous osteocartilaginous cap to treat overgrowth, based on the observation that overgrowth did not occur following disarticulation (9). Their objective was to create a stump similar to a disarticulation by using a diaphyseal amputation. Since then, variations of their technique have emerged, using the proximal part of the fibula, the iliac crest, or various tarsal bones as sources for the osteocartilaginous cap (1). Fedorak et al. also reported a low revision rate of 10% when using ipsilateral fibular transplantation to the tibia (11). In addition to capping methods, distal tibiofibular synostosis has been explored as a simple and safe technique. However, Drvaric et al. reported that this procedure is ineffective due to a high rate of recurrent overgrowth (6).

Based on the cases and the successful outcomes using autologous fibula capping, it appears that this method is indeed a safe and effective approach for preventing stump overgrowth. Harvesting the non-vascularized ipsilateral proximal fibula as an autograft eliminates the need for an additional surgical donor site, simplifying the procedure. Detaching the insertion of lateral collateral ligament (LCL) on the fibula did not cause knee instability in our patients. Similarly, Fedorak et al. also did not repair the LCL in his 47 cases of osteocartiliginous transfer (11). Only one patient had persistent valgus instability, which was managed with a thigh lacer. Another structure that was released from the fibula head in our patients was the distal insertion of the biceps femoris tendon (BFT), which was sutured together with LCL to the surrounding soft tissue. This did not appear to affect the knee flexion significantly, most likely due to attachment to the tibial condyle as well as medial hamstring muscle insertions were still intact.

Apart from that, the interference fit between the fibula head and tibial diaphysis provides stability without requiring additional fixation, further enhancing the feasibility and convenience of the technique. Considering the high prevalence of stump overgrowth and its associated complications in children, we found incorporating autologous fibula capping at the time of primary amputation seems to be a proactive measure to address the issue. By implementing this technique early on, the risk of subsequent stump overgrowth and the need for multiple revision surgeries can be minimized.

## Data Availability

The original contributions presented in the study are included in the article/Supplementary Material, further inquiries can be directed to the corresponding author.
